# Effective Limb Salvage Using a Distally Based Peroneus Brevis Flap for Distal Tibial Osteomyelitis

**DOI:** 10.7759/cureus.98065

**Published:** 2025-11-29

**Authors:** Amena F Almubarak, Jenna A Almannaei, Qasim Alnahawi, Mokhtar Mahmoud Abdelhamid

**Affiliations:** 1 Department of General Surgery, King Hamad University Hospital, Muharraq, BHR; 2 College of Medicine, Dar Al Uloom University, Riyadh, SAU; 3 Department of Plastic Surgery, King Hamad University Hospital, Muharraq, BHR

**Keywords:** bone graft, chronic osteomyelitis, distal tibia, limb salvage, peroneus brevis flap, soft tissue coverage

## Abstract

Post-traumatic osteomyelitis of the distal third of the tibia represents a significant reconstructive challenge due to limited soft-tissue coverage, poor vascularity, and a high risk of recurrent infection. We present the case of a 60-year-old diabetic male patient who developed chronic osteomyelitis following traumatic injury to the lower third of his leg. The patient underwent staged management, including radical debridement, culture-directed antibiotic therapy, autologous cancellous bone grafting, and soft-tissue reconstruction with a distally based peroneus brevis muscle flap. Complete wound healing and full restoration of limb function were achieved without recurrence of infection. This case underscores the effectiveness of the peroneus brevis flap as a reliable, technically straightforward, and resource-efficient alternative to microsurgical free flaps for distal tibial defects. When combined with thorough infection control and skeletal reconstruction, this approach provides a practical and successful limb-salvage solution for chronic distal tibial osteomyelitis.

## Introduction

Post-traumatic osteomyelitis occurs in 1-2% of closed fractures and 10-30% of open fractures. In severe Gustilo type III tibial injuries, the rate can be as high as 50%. These high infection rates highlight the importance of reliable soft-tissue reconstruction methods for distal tibial defects, which are considered a significant reconstructive challenge. Limited vascularity, inadequate soft-tissue coverage, and comorbidities such as diabetes mellitus (DM) impair healing and increase the risk of recurrent infection. Limb salvage in these circumstances typically requires an integrated approach that combines infection control, skeletal reconstruction, and durable soft-tissue coverage [1-3].

Local muscle flaps, including the distally based peroneus brevis muscle flap (PBMF), provide reliable vascularized coverage for distal leg defects with minimal donor-site morbidity and without the need for microsurgical expertise [3]. In this case, the PBMF was effective in combination with both bone grafting and external fixation to achieve soft-tissue coverage and infection control.

## Case presentation

**Figure 1:**
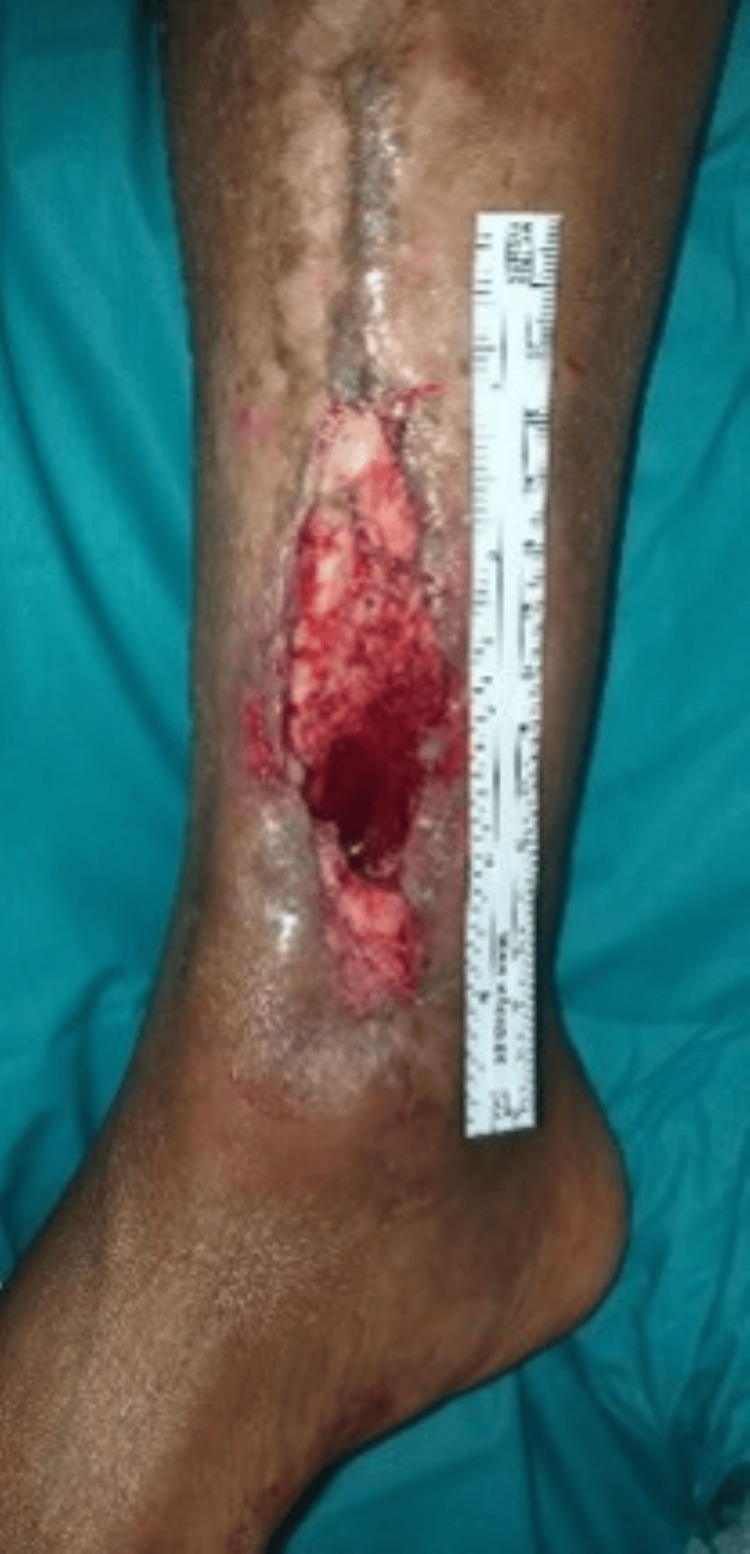
Post-incision and drainage

A 60-year-old Bahraini man with type 2 DM, hypertension, and dyslipidemia presented to the emergency department in 2020 with purulent discharge from a wound over the distal right tibia. He had a history of open reduction and internal fixation with plating for a distal tibial fracture in 2008. Physical examination revealed a 1 × 3 cm wound on the anteromedial aspect of the distal tibia, which was initially debrided in the ED (Figure 1).

**Figure 2:**
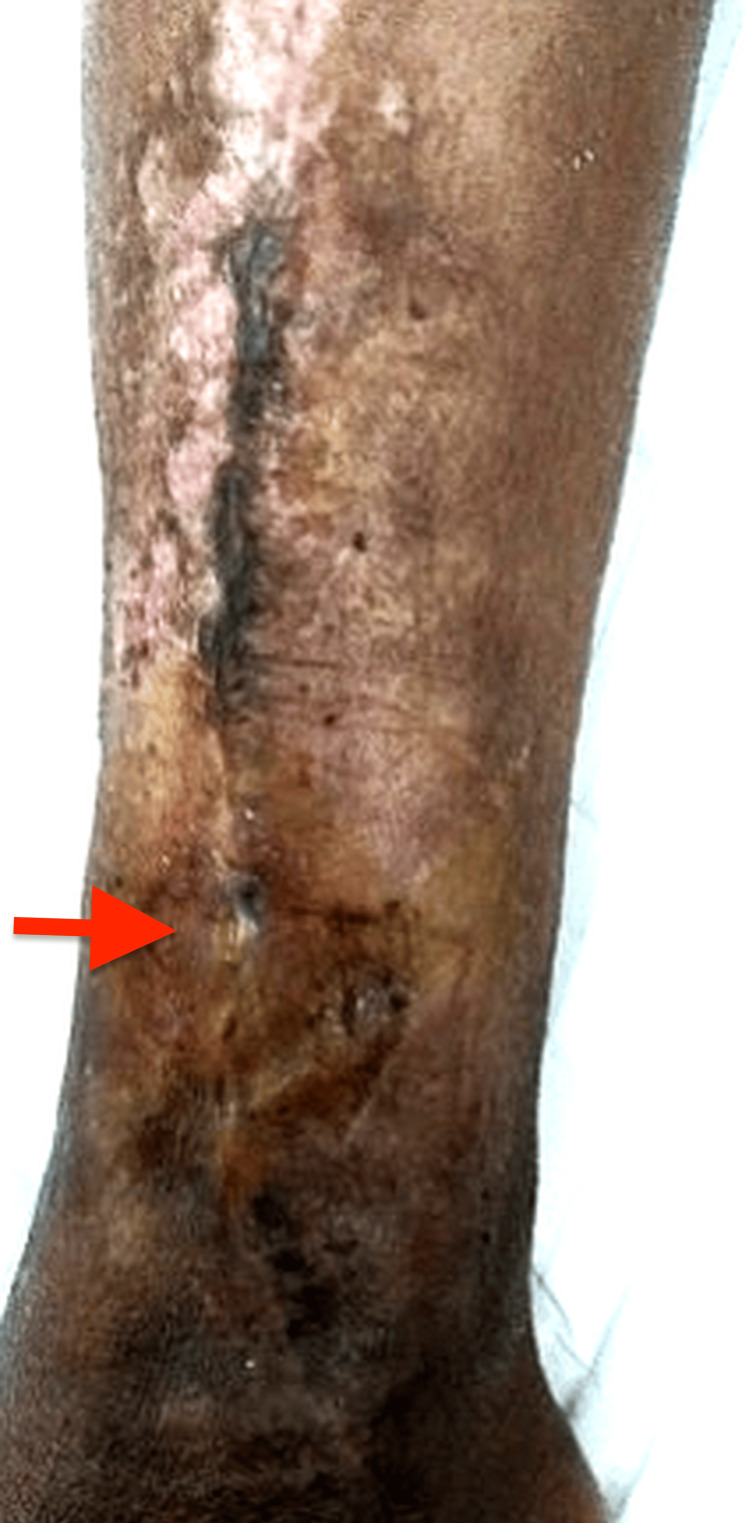
Sinus tract detected over the lower third of the leg

**Figure 3:**
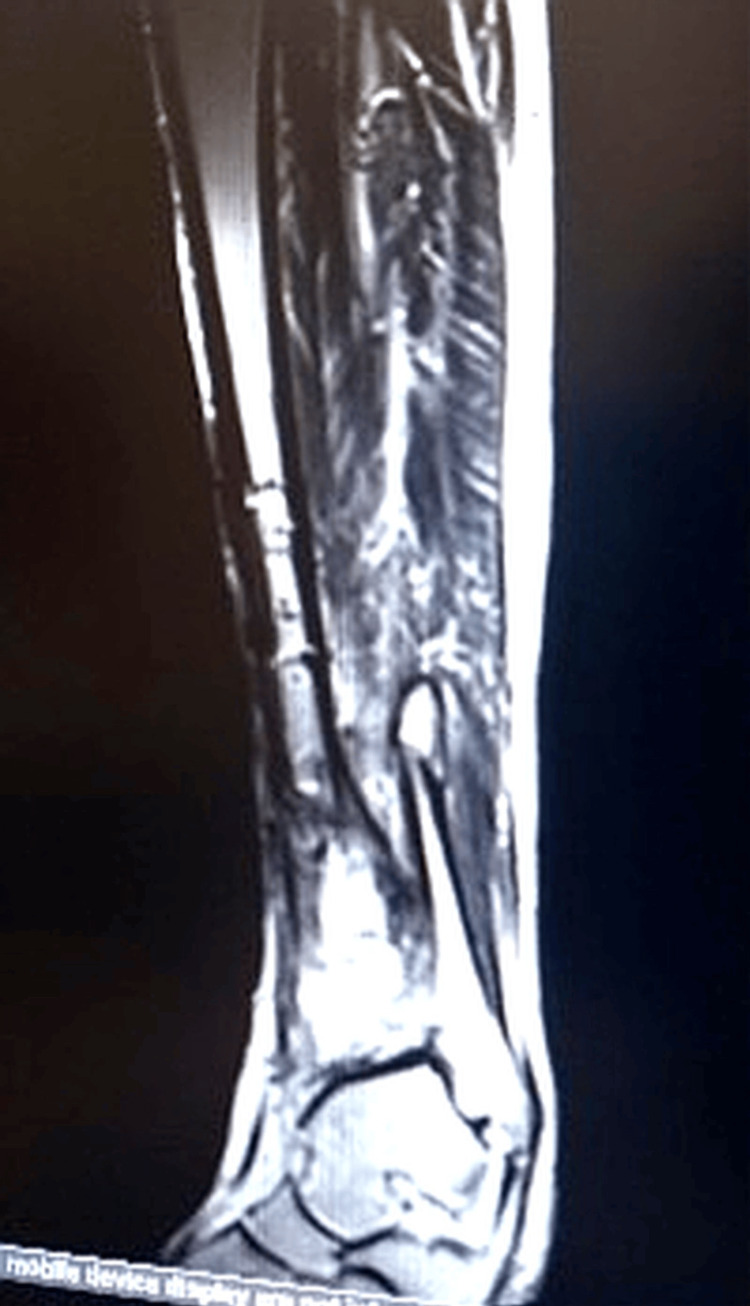
MRI demonstrating osteomyelitis with sequestrum

During follow-up in the plastic surgery clinic, a sinus tract was identified over the distal third of the leg, and MRI demonstrated features of active osteomyelitis with sequestrum formation (Figure 2, Figure 3). Initial wound cultures grew *Klebsiella pneumoniae*, while intraoperative tissue cultures isolated *Staphylococcus aureus*, considered more reliable for guiding therapy.

**Figure 4:**
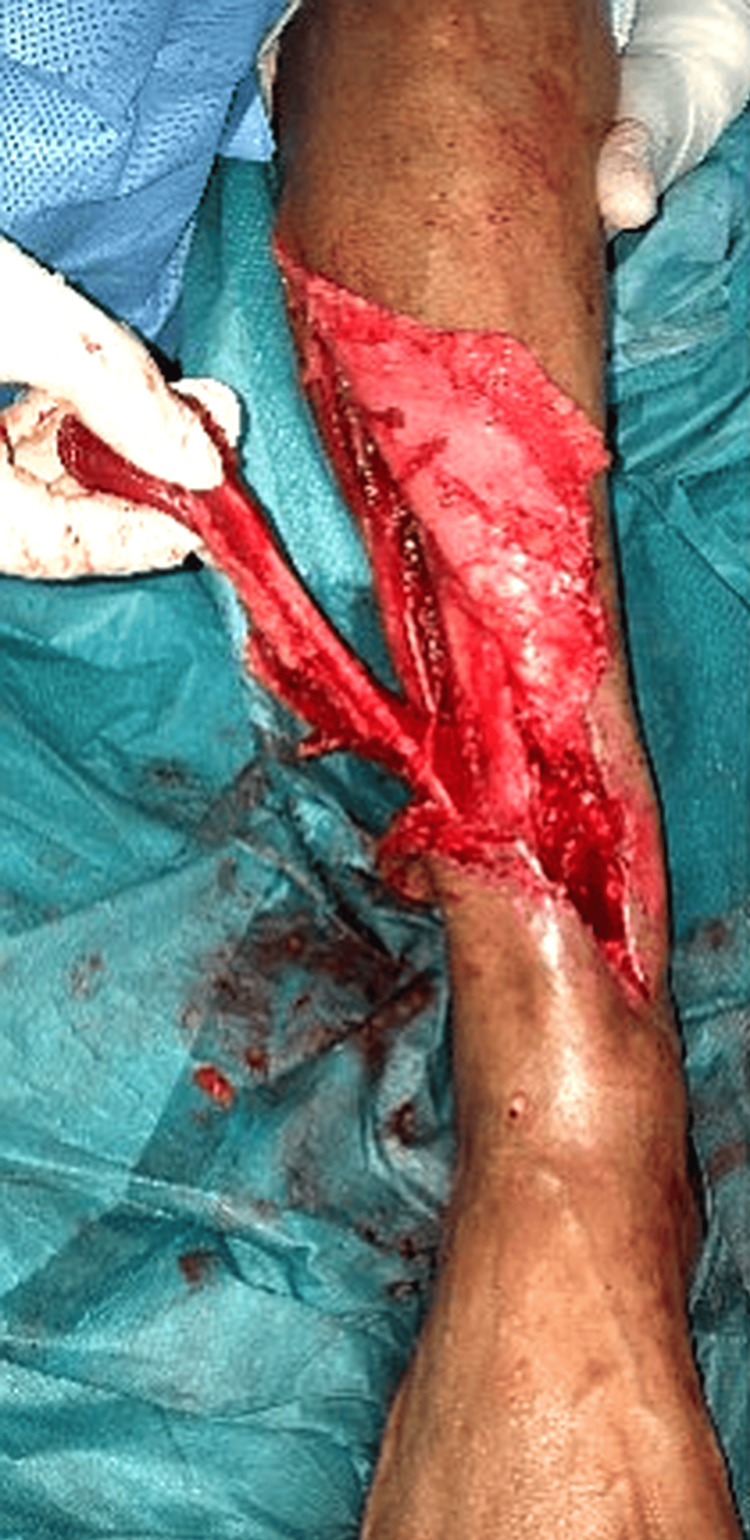
Radical debridement

**Figure 5:**
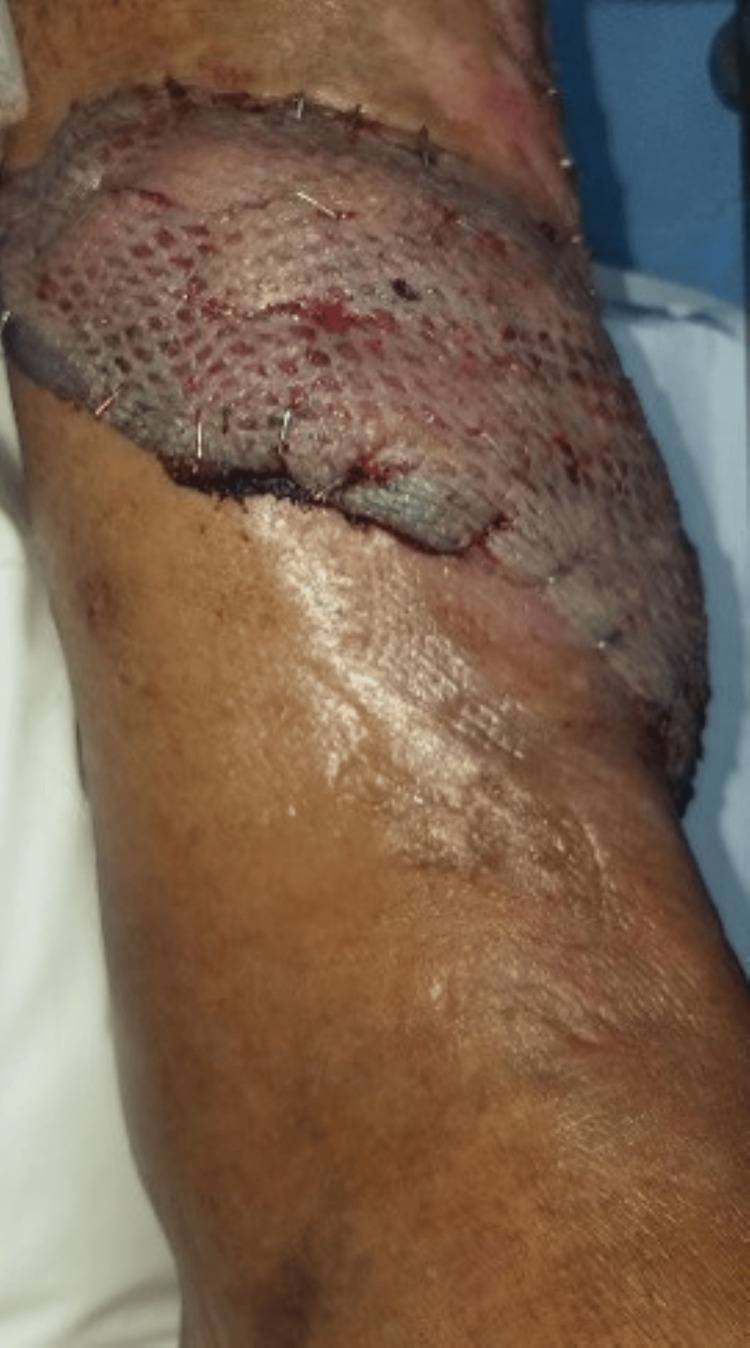
Soft-tissue coverage using distally based PBMF and STSG PBMF, peroneus brevis muscle flap; STSG, split-thickness skin graft

**Figure 6:**
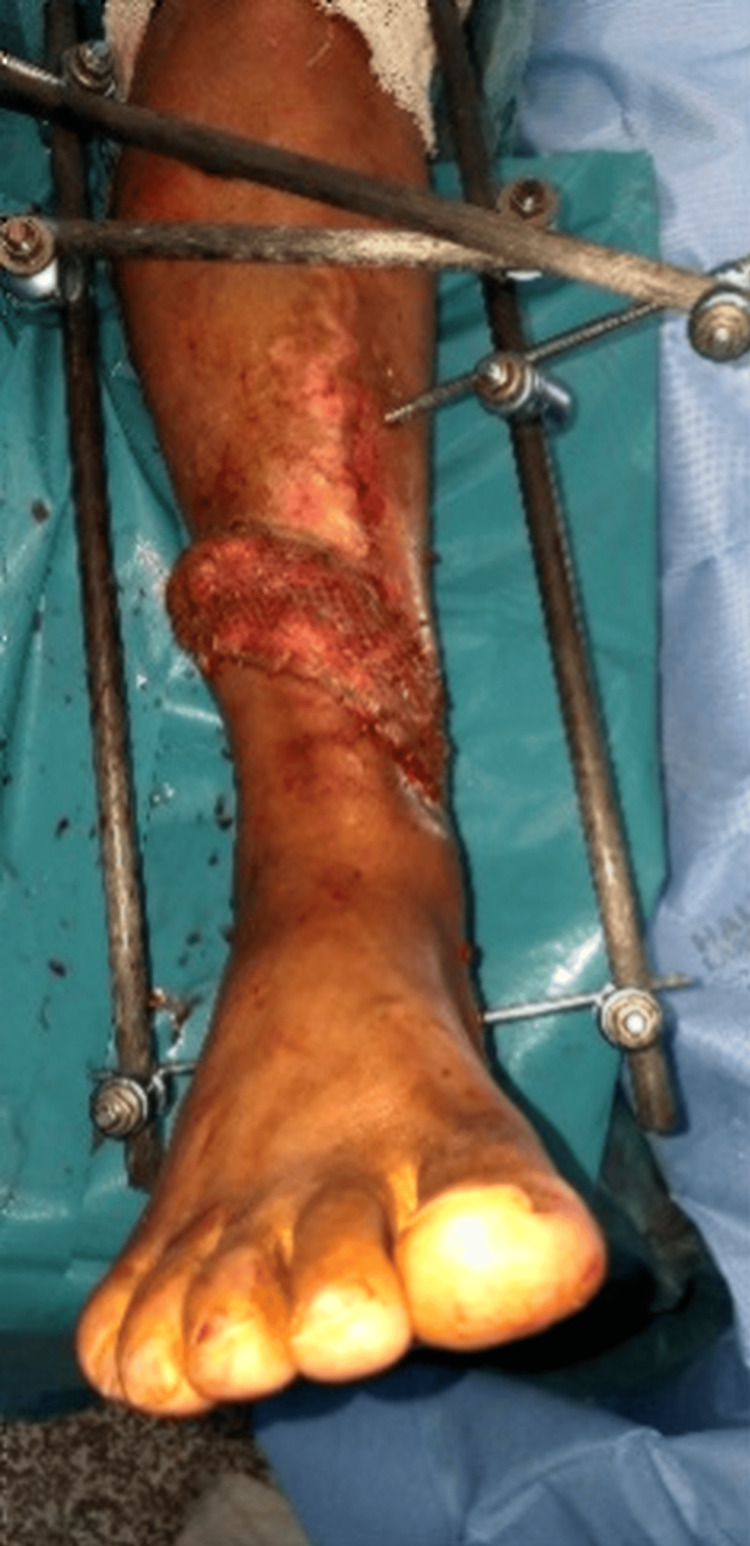
Ilizarov fixator placement

**Figure 7:**
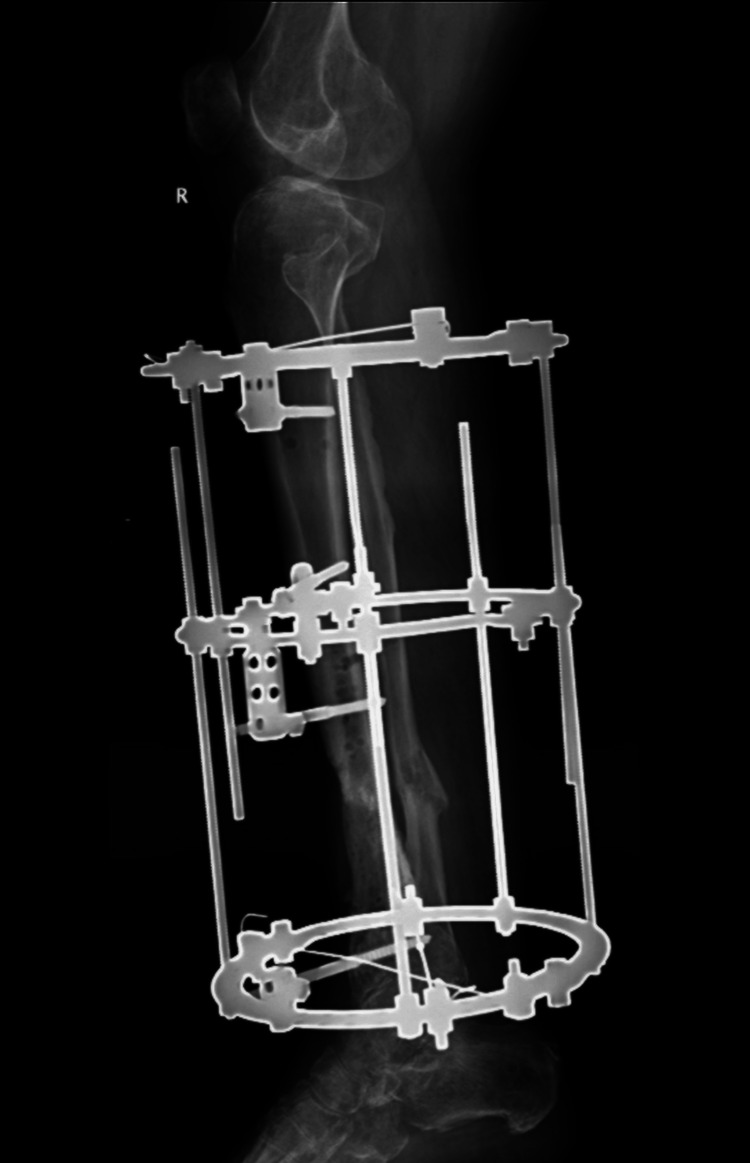
X-ray during follow-up

**Figure 8:**
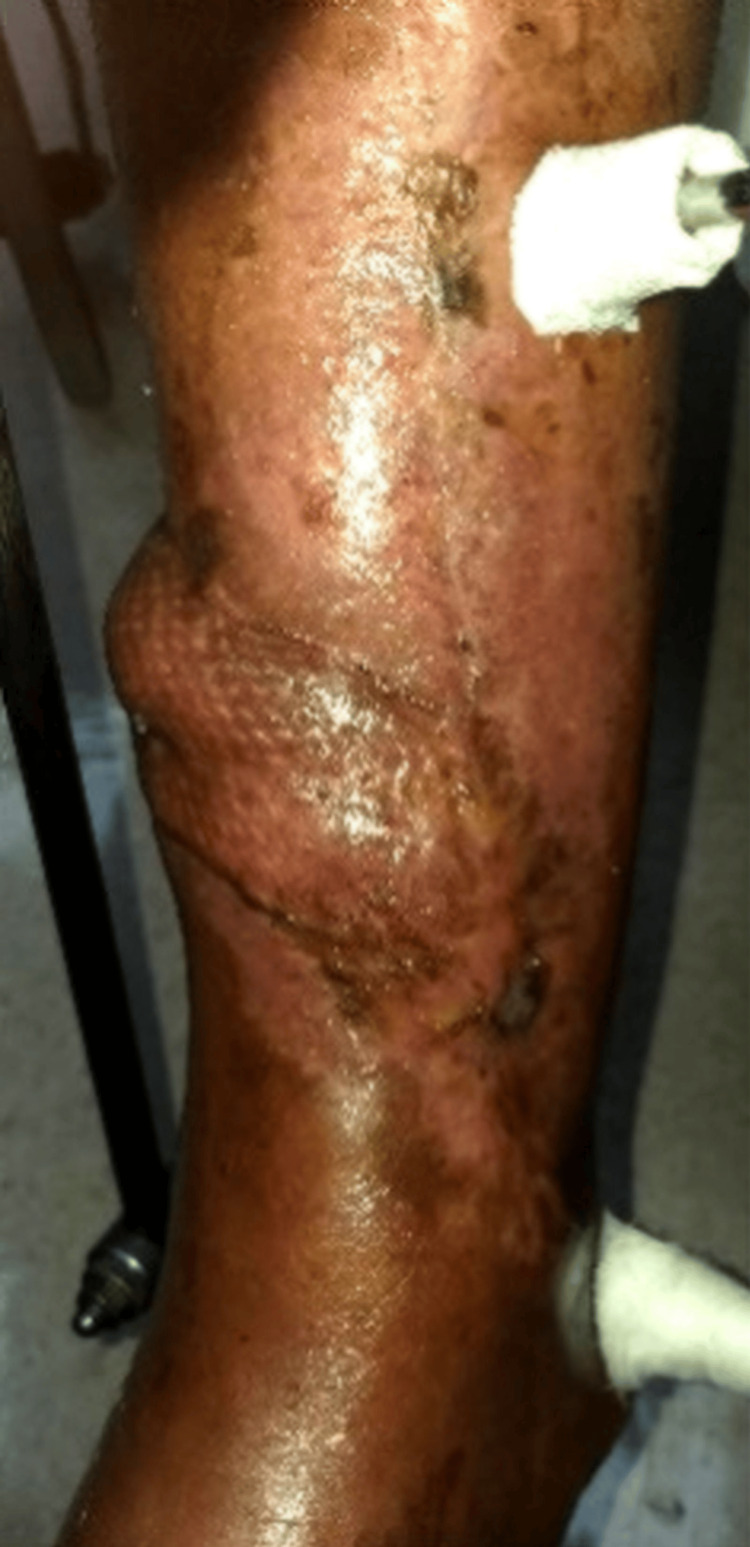
Follow-up evaluation

**Figure 9:**
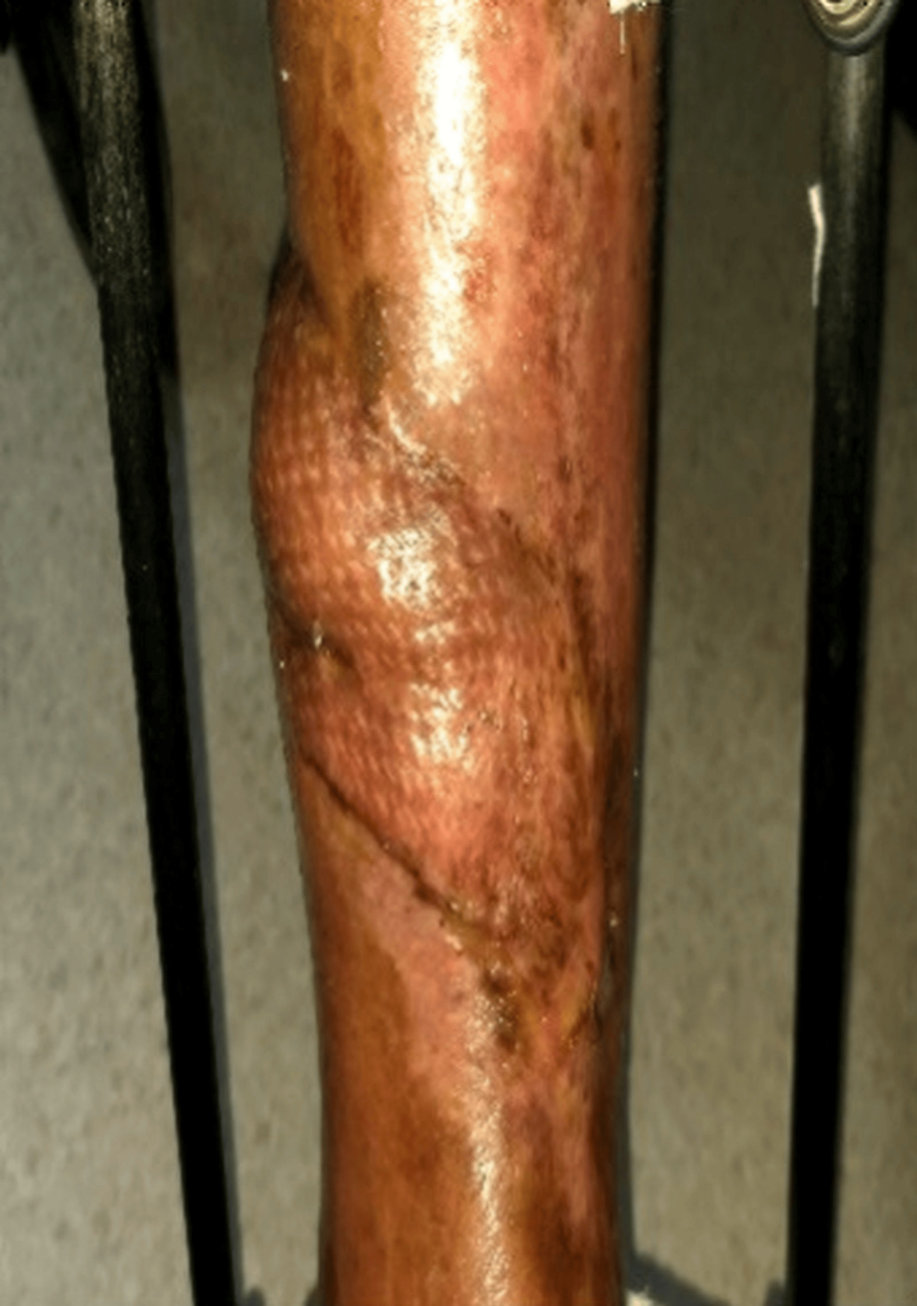
Six-week follow-up showing continued healing and stability

The patient underwent staged management (Table 1). Radical debridement of necrotic soft tissue and bone, removal of hardware, and insertion of antibiotic beads established a sterile, vascularized wound bed (Figure 4). Soft-tissue coverage was achieved using a distally based PBMF with a split-thickness skin graft (STSG) (Figure 5). Limb stabilization and reconstruction of a ~5 cm tibial defect were performed using iliac crest autologous cancellous bone grafting and application of an Ilizarov circular external fixator (Figure 6, Figure 7). Follow-up evaluations demonstrated progressive wound healing and flap viability (Figure 8). At six weeks, the distal tibia showed continued healing and structural stability, with full weight-bearing achieved and no recurrence of infection at one-year follow-up (Figure 9).

**Table 1:** Surgical timeline PBMF, peroneus brevis muscle flap; STSG, split-thickness skin graft

Date	Status	Intervention	Outcome
February 5, 2020	Purulent discharge from the lower third of the tibia; infected hardware. Initial culture positive for *Klebsiella pneumoniae*	Incision and drainage of the abscess	Infection burden reduced
February 20, 2020	Sinus tract at the distal third of the tibia. MRI consistent with chronic osteomyelitis	Radical debridement of necrotic soft tissue and bone; removal of plate and screws; insertion of antibiotic beads. Based on the tissue culture and antibiotic sensitivity pattern, intravenous ceftriaxone for six weeks, followed by oral ciprofloxacin for four weeks	Necrotic tissue removed; hardware removed; vascular wound bed established with targeted local and systemic therapy for infection control
February 20, 2020	Coverage	Soft-tissue reconstruction using a distally based PBMF with STSG under Doppler guidance	Flap viable with robust perfusion and no necrosis; durable soft-tissue envelope achieved
April 15, 2020	Follow-up	99mTc-besilesomab leukocyte scan (LeukoScan)	No proximal spread of osteomyelitis; tracer uptake localized to the debrided distal tibial region
May 20, 2020	Stabilization and reconstruction	Removal of antibiotic beads; iliac crest autologous cancellous bone grafting for a ~5 cm defect; application of Ilizarov circular external fixator	Graft incorporated well; alignment maintained; early mobilization initiated
November 30, 2020	Final follow-up	Removal of the Ilizarov frame	Full weight-bearing achieved; no recurrence of infection at one-year follow-up

## Discussion

Chronic osteomyelitis of the distal tibia presents a major management challenge, particularly in patients with DM, who are more susceptible to infection and delayed healing. The main challenges include inadequate vascularity, limited soft-tissue coverage, and proximity of infection to bony prominences. Effective management requires a staged, multidisciplinary approach incorporating thorough debridement, skeletal stabilization, and soft-tissue reconstruction [1,4-6]. In this case, treatment consisted of radical debridement, local antibiotic bead implantation, soft-tissue coverage using a distally based PBMF, followed by cancellous bone grafting with Ilizarov external fixation [6].

The cornerstone of treatment is aggressive debridement until healthy, bleeding bone is encountered, a finding commonly referred to as the paprika sign. Antibiotic beads provide high local antibiotic concentrations while temporarily occupying dead space. This step is critical for breaking the cycle of infection and preparing the wound for reconstruction [6]. Muscle flaps play a pivotal role in managing infected wounds by enhancing blood flow, improving antibiotic delivery, and removing necrotic tissue, all of which promote infection resolution and tissue healing [6,7]. The PBMF is particularly suitable for small to medium defects in the distal leg, relying on perforators from the peroneal artery and allowing harvest without the need for microvascular anastomosis [3,5,8-10].

Although free flaps are considered the gold standard for extensive or proximal limb defects, they require microsurgical expertise, longer operative time, and specialized postoperative monitoring [4,11-15]. In contrast, the PBMF offers a low-resource, high-yield alternative that is reproducible across different surgical settings and is especially appropriate for elderly or comorbid patients [3,5,10]. The peroneus brevis muscle originates from the distal two-thirds of the fibula and inserts at the base of the fifth metatarsal. Its segmental blood supply arises from perforators of the peroneal artery, allowing its use as a reverse (distally based) flap [2,5,7,8]. In this configuration, the flap pivots on distal peroneal perforators, typically located 6-8 cm above the lateral malleolus, allowing coverage of defects in the distal third of the leg, ankle, and hindfoot [3,5,8,12].

In this case, the PBMF provided several advantages. It did not require microvascular anastomosis, making it suitable for resource-limited environments and high-risk patients [6,7,10]. The major vessels were preserved, maintaining the integrity of key arterial and muscular structures [3]. Consistent vascular anatomy, confirmed with Doppler, ensured reliability even in patients with vascular compromise [2,5,8-15]. Additionally, the flap exhibited a progressive auto-thinning effect, providing good contour and cosmetic outcomes without the need for secondary debulking [5,13]. Preoperative Doppler ultrasound confirmed adequate distal perforators from the peroneal artery. The flap was elevated through a standard lateral approach, reversed distally, and inset to cover the tibial defect. An STSG was applied over the muscle. The flap remained viable with no ischemic changes, demonstrating its reliability and adaptability in diabetic patients.

Compared to the reverse sural artery flap, which is also used for distal leg reconstruction, the distally based PBMF offers several advantages [5,12,13]. It reduces venous congestion due to muscular venous drainage rather than reliance on a subcutaneous pedicle. The tissue is thinner and more pliable, making it suitable for covering bony surfaces such as the tibia or malleolus [13,14]. The flap also provides superior infection control due to better perfusion and antibiotic delivery by muscle tissue [6,7,10]. Lower complication rates have been reported in diabetic patients, with success rates above 80% in studies by Sahu et al. (2019) and Hassan et al. (2020) [5,11]. While free flaps remain preferable for large or complex defects, they require advanced microsurgical facilities and longer operative times. In contrast, the PBMF provides reliable coverage with less operative complexity, faster recovery, and lower risk in high-risk or elderly patients.

Following soft-tissue stabilization, cancellous bone grafting from the iliac crest was performed to address the 5-cm post-debridement tibial defect. Ilizarov external fixation provided stable alignment while avoiding the introduction of internal hardware into a previously infected site [1,6,11,16,17]. This method promotes early mobilization, functional rehabilitation, and osteogenesis through controlled mechanical loading. Autologous grafts remain the gold standard in infected settings because they provide both osteoconductive and osteoinductive properties. Although synthetic substitutes are under investigation, their performance in contaminated environments remains suboptimal.

A summary of relevant literature is provided in Table 2.

**Table 2:** Literature review CAD, coronary artery disease; DM, diabetes mellitus; HTN, hypertension; PBMF, peroneus brevis muscle flap; PVD, peripheral vascular disease

Study	Patients (n)	Flap type	Complications	Flap loss	Success rate	Follow-up	Comorbidities
Eren et al. (2001) [3]	19	Distally based PBMF	Minor wound breakdowns	0	84%	6-72 months	Lower limb trauma
Sahu et al. (2019) [5]	25	Distally based PBMF	Five cases of marginal necrosis	0	80%	6-12 months	DM, trauma
Antonini et al. (2017) [6]	11	Distally based PBMF	Partial necrosis (minor)	0	91%	36-52 months	Diabetic limb salvage
Yang et al. (2005) [8]	6	Distally based PBMF	One graft failure, two partial losses	0	83%	Not reported	Not reported
Bach et al. (2007) [9]	15	Distally based PBMF	One superficial necrosis (minor)	0	100%	6-13 months	Osteomyelitis, vascular risks
Nava et al. (2024) [10]	10	Distally based PBMF	One hematoma, one recurrence	0	80%	1-29 months	Bone infection
Hassan et al. (2020) [11]	20	Distally based PBMF	Two cases of patient dissatisfaction	0	90%	6 months	DM, PVD
Vaghani et al. (2021) [12]	10	Distally based PBMF	One case of marginal necrosis	0	90%	6 months	Trauma
Thammannagowda et al. (2014) [13]	32	Distally based PBMF	Five tip necrosis, two partial losses	0	94%	6 months	Diabetic ulcer
Rao et al. (2022) [14]	6	Distally based PBMF	Three cases of superficial necrosis	0	100%	3-6 months	Trauma, DM, CAD
Ng et al. (2010) [15]	5	Distally based PBMF	One distal necrosis, two skin graft failures	0	100%	6-12 months	DM, HTN
Rodriguez Collazo et al. (2013) [16]	1	Distally based PBMF	None	0	100%	Healed at 4 weeks	Frail
Bischoff et al. (2023) [17]	1	Distally based PBMF	Partial compromise	0	100%	32 months	Neuro-ischemic ulcer

Early soft-tissue reconstruction, ideally within 72 hours after debridement, optimizes outcomes [4]. Preoperative Doppler assessment is essential for flap planning, particularly in patients with vascular comorbidities [12-14]. A comprehensive approach that combines the distally based PBMF with autologous bone grafting and stable external fixation ensures optimal infection control and limb preservation [14,16]. The PBMF is a reliable option for distal leg soft-tissue defects, providing dependable vascularity and minimal donor-site morbidity. When integrated into a staged approach with debridement, targeted antibiotics, and Ilizarov stabilization, it supports effective infection control and successful limb preservation [4-8].

## Conclusions

Chronic osteomyelitis of the distal third of the tibia presents a complex reconstructive challenge due to the high risk of recurrent infection, limited soft-tissue coverage, and poor vascularity. We present the case of a 60-year-old patient with DM who developed chronic osteomyelitis following tibial hardware implantation. The patient underwent staged management, including radical debridement, culture-directed antibiotic therapy, autologous cancellous bone grafting, and soft-tissue reconstruction using a distally based PBMF. Complete wound healing and full restoration of limb function were achieved without recurrence of infection.

This case highlights the PBMF as a resource-efficient, technically straightforward, and reliable alternative to microsurgical free flaps for distal tibial defects, particularly in high-risk or resource-limited settings. The combination of local flap coverage, bone reconstruction, and meticulous infection control provides an effective limb-salvage strategy for chronic distal tibial osteomyelitis.
